# Toward clinical understanding of aristolochic acid upper-tract urothelial carcinoma

**DOI:** 10.7150/thno.46489

**Published:** 2020-04-21

**Authors:** Arnoud Boot, Nanhai Jiang, Steven G. Rozen

**Affiliations:** 1 Duke-NUS Centre for Computational Biology,Duke-NUS Medical School, 8 College Road, 169857 Singapore; 2 Signature Research Programme in Cancer & Stem Cell Biology, Duke-NUS Medical School, 8 College Road, 169857 Singapore; 3 SingHealth Duke-NUS Institute of Precision Medicine, 5 Hospital Drive 169609 Singapore; 4 SingHealth Duke-NUS Global Health Institute, 8 College Road, 169857 Singapore

## Abstract

A cluster of patients poisoned by herbal medicine in the 1990s revealed that aristolochic acid (AA) causes kidney failure and upper tract urothelial carcinoma (UTUC). Recent research demonstrated that this was not an isolated incident; on the contrary, AA exposure is widespread in East Asia. This editorial highlights research by Lu and colleagues that investigates clinical characteristics of AA and non-AA UTUCs from 90 patients in Beijing based on the AA mutational signature. The study also detected AA mutations in non-tumor tissue of AA exposed patients and showed that AA mutations can be detected in urine, which might form the basis for non-invasive tests for AA exposure.

Follow up of patients from a herbal-medicine-poisoning cluster in a Belgian weight-loss clinic revealed that aristolochic acid (AA) exposure often causes upper tract urothelial carcinomas (UTUCs) - cancers in the ureter or renal pelvis 1, 2. Nevertheless, despite concerns that AA exposure might be widespread 3, 4, until recently, AA-induced cancers were studied mainly in two geographic hot spots - the Balkans and Taiwan 5-14. In the last few years, however, research using AA's DNA-damage footprint - its mutational signature - revealed widespread AA exposure in East Asia 8, 15-20. Furthermore, mutational signatures also implicated AA in cancer types in addition to UTUC: cancers of the kidney, bladder, bile duct, and liver.

Because these discoveries are recent, as yet there has been little study of the clinical characteristics or of the epidemiology of AA cancers; this includes AA UTUC, even though this was the first type of cancer linked to AA exposure. The study by Lu and colleagues, being the first study based on the AA mutational signature, marks an important step forward in this area 21.

Lu and colleagues reported that 62 of the 90 patients studied have the AA mutational signature. This number seems approximately correct; a more conservative estimate is that 59 patients were exposed to AA (mSigAct signature presence test and Benjamini-Hochberg false discovery rate < 0.05) 15. The “AA Sig Subtype” tumors in Figure 1A of 21 do not include all AA-mutagenized tumors, because some tumors with clear AA mutations nevertheless clustered away from the tumors dominated by AA (e.g. tumor T013). Nevertheless, the AA Sig Subtype classification provides a reasonable dichotomization between tumors with high AA exposures and those with low or no AA exposure.

Lu and colleagues' data allow a rough estimate of the overall prevalence of AA exposure, even though the study deliberately enriched for patients with self-reported AA exposure. Over all the data (Supplemental Figure S1 in 21), ~11.5% of the patients reported AA exposure. Using the conservative estimates of AA exposure (not only the AA Sig Subtype tumors) 23 / 27 patients with self-reported exposure had the AA signature, and 36 / 63 patients with no reported exposure had the signature (Supplementary Files 1 and 2 in 21). We can estimate prevalence of even a low level of AA exposure in this population as (23 / 27) x 11.5 % + (36 / 63) x 88.5% = 60%. The discordance between self-reported exposure and presence of the mutational signature is to be expected. It stems from the bewildering variety of herbal formulations, the frequency of misidentification of herbs, the varying mutagenicity of different AA-related compounds, and the widely varying concentrations of AA-related compounds in different samples of the same herb 22-26. In contrast to previous studies, assessing exposure by the AA mutational signature is a strength of the paper by Lu and colleagues.

Lu and colleagues observed correlations between the AA Sig Subtype and several clinical factors. The AA Sig Subtype was strongly associated with worse kidney function, which is consistent with the known association of AA exposure with both kidney failure and UTUC. The AA Sig Subtype was also more prevalent in women, in patients with multifocal tumors, in tumors of the renal pelvis as opposed to the ureter, and in tumors of lower stage. Many, but not all of these findings were consistent with previous results that were not based on the AA mutational signature 11, 27, 28.

Notably, Lu and colleagues reported that the AA Sig Subtype tumors had better survival, a finding previously reported by Zhong and colleagues, but not by two earlier studies 11, 27, 28. Multivariate analysis of Lu and colleagues' data indicates that this is primarily due to the association of AA Sig Subtype tumors with lower tumor stage. To our knowledge, the reason that AA exposure is associated with lower tumor stage has not been studied.

Lu and colleagues also found that the AA Sig Subtype tumors had high numbers of in-silico-predicted neoantigens, and thus might be promising candidates for immunotherapy. Supporting this, AA Sig Subtype tumors had higher levels of infiltrating immune cells. The study also provided extensive confirmation that the AA mutational signature can be detected in non-malignant urothelial tissues 29.

Importantly, Lu and colleagues showed that the AA mutational signature can be detected in cell-free DNA in urine. Given that AA exposure is widespread, noninvasive tests for AA exposure would be highly useful. First, noninvasive tests could be used to study the epidemiology of AA associated cancers, since, as Lu and colleagues' study confirms, it is difficult to estimate AA exposure from retrospective histories. Second, noninvasive tests might also be useful for the clinical management of AA cancers. Third, the tests might be useful for the clinical management of AA exposed individuals, for whom testing positive for AA-exposure might be an indication for regular screening for AA-associated diseases such as kidney failure and cancers of the urinary tract, liver and bile-duct.

In summary, to our knowledge, this is the first study to investigate the clinical characteristics of AA UTUCs using AA exposure assessed by the AA mutational signature. Further studies of the clinical characteristics of AA cancers (UTUC and other cancer types as well) and of the epidemiology of AA exposure should be performed. In particular, the reason that AA UTUCs tend to have lower tumor stage needs to be investigated. Finally, a noninvasive test to detect AA mutagenesis and thereby more accurately assess previous exposure would be a large advance. Consequently, the promising finding that the AA signature can be detected in urine needs to be pursued with the aim of developing a test to detect the presence and level of AA mutations -- one that would be usable in terms of cost, robustness, sensitivity, and positive predictive value.

## Abbreviations

AA: aristolochic acid
UTUC: upper tract urothelial carcinoma
